# Exploring the Binding Interaction of Raf Kinase Inhibitory Protein With the N-Terminal of C-Raf Through Molecular Docking and Molecular Dynamics Simulation

**DOI:** 10.3389/fmolb.2021.655035

**Published:** 2021-05-28

**Authors:** Shraddha Parate, Shailima Rampogu, Gihwan Lee, Jong Chan Hong, Keun Woo Lee

**Affiliations:** ^1^ Division of Life Sciences, Division of Applied Life Science (BK21 Plus), Plant Molecular Biology and Biotechnology Research Center (PMBBRC), Research Institute of Natural Science (RINS), Gyeongsang National University (GNU), Jinju, Korea; ^2^ Division of Life Sciences, Plant Molecular Biology and Biotechnology Research Center (PMBBRC), Department of Bio and Medical Big Data (BK21 Four Program), Research Institute of Natural Science (RINS), Gyeongsang National University (GNU), Jinju, Korea

**Keywords:** RKIP, C-Raf, protein-protein docking, HADDOCK, ZDOCK, molecular dynamics simulation, MM/PBSA, binding sites prediction

## Abstract

Protein-protein interactions are indispensable physiological processes regulating several biological functions. Despite the availability of structural information on protein-protein complexes, deciphering their complex topology remains an outstanding challenge. Raf kinase inhibitory protein (RKIP) has gained substantial attention as a favorable molecular target for numerous pathologies including cancer and Alzheimer’s disease. RKIP interferes with the RAF/MEK/ERK signaling cascade by endogenously binding with C-Raf (Raf-1 kinase) and preventing its activation. In the current investigation, the binding of RKIP with C-Raf was explored by knowledge-based protein-protein docking web-servers including HADDOCK and ZDOCK and a consensus binding mode of C-Raf/RKIP structural complex was obtained. Molecular dynamics (MD) simulations were further performed in an explicit solvent to sample the conformations for when RKIP binds to C-Raf. Some of the conserved interface residues were mutated to alanine, phenylalanine and leucine and the impact of mutations was estimated by additional MD simulations and MM/PBSA analysis for the wild-type (WT) and constructed mutant complexes. Substantial decrease in binding free energy was observed for the mutant complexes as compared to the binding free energy of WT C-Raf/RKIP structural complex. Furthermore, a considerable increase in average backbone root mean square deviation and fluctuation was perceived for the mutant complexes. Moreover, per-residue energy contribution analysis of the equilibrated simulation trajectory by HawkDock and ANCHOR web-servers was conducted to characterize the key residues for the complex formation. One residue each from C-Raf (Arg398) and RKIP (Lys80) were identified as the druggable “hot spots” constituting the core of the binding interface and corroborated by additional long-time scale (300 ns) MD simulation of Arg398Ala mutant complex. A notable conformational change in Arg398Ala mutant occurred near the mutation site as compared to the equilibrated C-Raf/RKIP native state conformation and an essential hydrogen bonding interaction was lost. The thirteen binding sites assimilated from the overall analysis were mapped onto the complex as surface and divided into active and allosteric binding sites, depending on their location at the interface. The acquired information on the predicted 3D structural complex and the detected sites aid as promising targets in designing novel inhibitors to block the C-Raf/RKIP interaction.

## Introduction

The physiological processes including signal transduction, cell proliferation, cell division, enzyme inhibition, and DNA repair are controlled via recognition and association of different proteins (Thiel et al., 2012). Nearly 6,50,000 protein-protein interactions (PPI) referred to as “interactome” regulate human life and dysregulation of any interaction leads to pathological conditions including neurological disorders and cancer (Ryan and Matthews, 2005; Stumpf et al., 2008; Sable and Jois, 2015; Ottmann, 2016). Despite the vast biological significance of protein-protein complexes, elucidating their structures and association mechanisms remains a notoriously challenging task (Zinzalla and Thurston, 2009; Ngounou Wetie et al., 2013). Protein-protein docking is a fundamental computational tool often combined with experimentally predicted information to decipher the association mechanism of such complexes (Ritchie, 2008; Kaczor et al., 2018). Innumerable protein-protein docking web-servers have been developed with diverse sampling algorithms and scoring functions in order to accurately predict the binding mode between two protein structures (Gromiha et al., 2017; Porter et al., 2019). Due to varying differences in their docking and scoring strategies, choosing an appropriate protocol for docking is a tricky problem in itself (Huang, 2014; Park et al., 2015; Gromiha et al., 2017; Porter et al., 2019). The CAPRI (Critical Assessment of PRedicted Interactions) community-wide effort attempts to dock the same proteins provided by the assessors in a scientific meeting held every six months for discussing protein-protein docking accuracy (Janin, 2010). This CAPRI meeting divides the innumerable protein-protein docking tools available into validated and non-validated ones (Kangueane et al., 2018). In addition to molecular docking, atomic-level molecular dynamics (MD) simulations characterize the structure, dynamics and stability of protein-protein complexes and provide an unprecedented sampling of the complexes formed by two protein monomeric structures (Kuroda and Gray, 2016; Shinobu et al., 2018).

Raf-1 kinase inhibitory protein (RKIP), also designated as phosphatidylethanolamine binding protein-1 (PEBP-1), is a 20–25 kDa evolutionarily conserved cytosolic protein that acts as a fundamental modulator of several signal transduction cascades including the mitogen-activated protein kinase (MAPK) pathway, G protein-coupled receptor (GPCR) pathway and the nuclear factor κB (NF-κB) pathway (Granovsky and Rosner, 2008; Zeng et al., 2008; Al-Mulla et al., 2013). RKIP was identified as an endogenous regulator of Raf-1 kinase (C-Raf), GPCR kinase 2 (GRK2) and kinases of the NF-κB pathway. In particular, RKIP interacts with cytoplasmic serine/threonine kinase Raf-1 and interferes with the Raf-1 dependent activation of MAPK/extracellular signal-regulated kinase (ERK) kinase (MEK), thereby disrupting the activation of ERK (Yeung et al., 2001; Hagan et al., 2006; Ribas et al., 2007; Vandamme et al., 2014). Pathophysiologically, dysregulated RKIP expression contributes to several malignancies (Keller et al., 2004; Klysik et al., 2008; Ling et al., 2014). Loss or reduced RKIP expression is associated with poor prognosis in numerous cancer types including endometrial cancer, cervical cancer, bladder cancer, breast cancer, gastric cancer, thyroid cancer and gliomas (Eun et al., 2010; Kim et al., 2010; Escara-Wilke et al., 2012; Martinho et al., 2012; Lamiman et al., 2014; Farooqi et al., 2015; Zaravinos et al., 2018). Downregulation of RKIP has also been observed in pathologies like diabetic nephropathy, sperm decapitation, heart failure, Alzheimer’s disease and lung-related pathologies (Keller et al., 2004; Klysik et al., 2008; Raquel-Cunha et al., 2019). RKIP plays a pivotal role as a predictive biomarker for various diseases making it a therapeutic target aimed at building probes that will potently and specifically perturb its function (Zeng et al., 2008; Yesilkanal and Rosner, 2018).

Elucidating the structural basis of RKIP binding with C-Raf is essential for completely understanding the regulation of C-Raf. Previous study by Trakul et al. reported that RKIP abrogates C-Raf activation by binding to its N-terminal region and inhibiting its phosphorylation at residues Ser338 and Tyr340/Tyr341 (Trakul et al., 2005). This data was consistent with another study by Park et al*.* where they investigated the binding of RKIP to C-Raf N-terminal region by mutational analysis. They substituted Ser338 with alanine (Ala) and Tyr340/Tyr341 with phenylalanine (Phe) and the mutation was observed to diminish the binding of RKIP with C-Raf (Park et al., 2006). A study published by Rath et al. examined the importance of the ligand-binding pocket of RKIP in binding with its substrate C-Raf at the aforementioned N-terminal region residues Ser338/Tyr340/Tyr341. The two highly conserved residues within the ligand-binding pocket of RKIP were mutated: Asp70 with Ala and Tyr120 with Phe. These RKIP mutants demonstrated diminishment in their capability to inhibit C-Raf, thereby establishing the significance of the ligand-binding pocket of RKIP in binding with its substrate (Rath et al., 2008). Wu et al*.* in a study published to further illuminate on the ligand-binding pocket of RKIP mutated seven residues to Ala (Asp70, Asp72, Tyr81, Glu83, Ser109, Tyr120, and Tyr181) and two residues to leucine (Leu) (Pro74 and Pro112). With the wild-type (WT) RKIP binding affinity of 154 mM^−1^ for C-Raf residues 1–147 amino acids, the binding affinity of mutants Pro74, Tyr81, Ser109, and Pro112 decreased by 30–50%. Furthermore, the binding affinity of Asp70, Glu83, and Tyr120 mutations considerably reduced to 30, 22, and 18 mM^−1^, respectively, while a sizable decrease was noticed in the affinity of mutations Asp72 (7 mM^−1^) and Tyr181 (3 mM^−1^) (Wu et al., 2014). Since RKIP binds to multiple regions of C-Raf, the mutations introduced by Wu et al*.* perturbed the binding of RKIP pocket residues with C-Raf residues (1–147 amino acids). However, the structural basis and interaction mode for binding of RKIP pocket residues with the N-terminal of C-Raf (340–615 amino acids) remains elusive in spite of the accessibility of their crystal structures. This opens the door for the prediction of their structural complex through molecular modeling techniques. Comprehending the binding mode of C-Raf/RKIP complex at the molecular level is of paramount importance for designing novel PPI inhibitors that could disrupt their association.

Herein, we propose a consensus mode of binding between the two proteins obtained through two knowledge-based protein-protein docking programs. Specifically, we carried out a reliable docking approach employing programs HADDOCK and ZDOCK combined with molecular dynamics (MD) simulations to investigate the C-Raf/RKIP PPI interface and uncover the interactions involved in the formation of their structural complex. Consequently, we introduced mutations at the conserved interface residues of the complex and carried out additional simulations to infer and compare their stabilities. Moreover, we evaluated free energy of binding for the complexes using MM/PBSA along with calculating per-residue energy decomposition using MM/GBSA. Subsequently, we also identified druggable “hot spots” that can be targeted for future drug optimization by ANCHOR web-server. An additional 300 ns of MD simulation was performed using the identified hot spot Arg398 as an exemplification to investigate the stability of the Arg398Ala mutant complex. The final aim was to shed some light on the residues involved in the complex formation and the identified sites for future designing of novel drugs. The complex predicted in this study is envisaged to be useful in procuring novel PPI inhibitors targeting the association of RKIP with the N-terminal of C-Raf.

## Materials and Methods

### Structure Retrieval and Refinement

The molecular details of RAF proto-oncogene serine/threonine protein kinase and its inhibitory protein RKIP/PEBP1 were retrieved from UniProtKB database with UniProt ID P04049 (RAF1_HUMAN) and P30086 (PEBP1_HUMAN), respectively, with proteins of lengths 307 (C-Raf) and 187 (RKIP) amino acids. Of the 18 X-ray diffraction structures available for C-Raf, nine structures were in the peptide form. Eight out of nine structures did not contain the desired residues (Tyr 340 and Tyr341) for PPI with RKIP and were present in the form of effector RAS binding domain (RBD). The X-ray diffraction structure (PDB ID: 3OMV) of resolution 4.00 Å with the presence of residues Tyr340 and Tyr341 was retrieved from the Research Collaboratory for Structural Bioinformatics Protein Data Bank (RCSB-PDB) with two identical chains: A and B (Hatzivassiliou et al., 2010). The structure was further evaluated in BIOVIA Discovery Studio (DS) Visualizer 2018 and chain B was eliminated along with the associated co-crystallized ligands of both chains. Similarly, the only three-dimensional structure of RKIP with the presence of C-Raf peptide ligand and a resolution of 1.95 Å was retrieved and downloaded from RCSB (PDB ID: 2QYQ) (Simister et al., 2011). The co-crystallized ligand O-phosphotyrosine was removed for further process of molecular docking, retaining only the crystal structure of RKIP. Structures of both the proteins were cleaned by Clean Protein protocol, missing loops were added and refined by minimization employing the CHARMM forcefield in DS. The minimized structures so obtained were employed for detailed PPI via two knowledge-based docking web-servers.

### Molecular Docking of RKIP With C-Raf

Protein docking is a quintessential tool used in molecular biology to identify key residues responsible for the interaction among two proteins (Kangueane et al., 2018). The binding interaction of RKIP with C-Raf was accomplished using two docking web-servers in order to achieve the best native conformation according to the knowledge of binding residues detected by aforementioned site-mutagenesis studies. These servers included HADDOCK 2.2 (Dominguez et al., 2003; De Vries et al., 2010; Van Zundert et al., 2016) and ZDOCK 3.0.2 (Pierce et al., 2011, Pierce et al., 2014). At this stage, one might remember that there are several web-servers for performing protein-protein docking and the results of different servers may not always be same. Therefore, with the intention of acquiring a consensus mode of binding, two aforementioned knowledge-based docking servers were utilized for the current study.

Knowledge-based protein-protein docking of C-Raf with RKIP was performed with the Easy interface of HADDOCK 2.2 (High Ambiguity-Driven biomolecular DOCKing) web-server (Van Zundert et al., 2016). HADDOCK (https://wenmr.science.uu.nl) considers experimental data to drive the process of molecular docking unlike ab initio docking protocols considering only the co-ordinates of the structures (van Dijk et al., 2006). The docking strategy followed by HADDOCK involves three steps including 1) randomization of orientations followed by energy minimization to remove steric clashes, 2) torsion angle dynamics utilizing torsion angles as degrees of freedom and 3) refinement with an explicit solvent in Cartesian space. Tyrosine (Tyr340 and Tyr341) residues of C-Raf and ligand-binding pocket residues of RKIP were mentioned as “active” residues involved in the intermolecular interaction, while “passive” residues were automatically defined as residues surrounding the active ones before submitting the docking job. The four clusters retrieved as HADDOCK results were probed for interactions between C-Raf and RKIP in DS.

ZDOCK (https://zdock.umassmed.edu/) facilitates global docking search on a 3D grid using the FFT algorithm via its user-friendly web interface combined with shape complementarity, electro statistics and statistical potential terms for scoring of the complex structures (Chen and Weng, 2002). ZDOCK version 3.0.2 was employed to perform the rigid-body docking of RKIP with C-Raf (Pierce et al., 2014). The tyrosines Tyr340 and Tyr341 of C-Raf and ligand-binding pocket residues of RKIP were selected as contacting residues for the docking process. The top 10 predictions of complex structures were downloaded as ZDOCK results and examined individually in DS by analyzing the interactions between C-Raf and RKIP.

The modeled structures from the above web-servers were analyzed and the output structures chosen as optimal ones from both servers were superimposed in DS to observe their alignment and comprehend the putative binding mode of interaction between C-Raf and its interacting partner RKIP. The intermolecular interactions between RKIP and C-Raf were analyzed by utilizing the Interaction Monitor implanted in DS.

### *In silico* Mutagenesis of Conserved Interface Residues

Given the structure of the complex, computational mutagenesis is extensively used to probe the protein-protein interfaces for “hot spot” residues affecting the binding affinity of the complex. In such cases, residues at the interface of WT complex structure are mutated and the binding affinity of the resulting complex is estimated (Massova and Kollman, 1999; Lise et al., 2009; Bradshaw et al., 2011). Some of the common residues acquired at the interface of HADDOCK and ZDOCK complex structures were mutated to Ala, Phe, and Leu. As the crystallographic structures of mutated proteins are not available, mutations were modelled using the Build and Edit Protein tool implemented in DS. Consequently, residues Tyr340 and Tyr341 of C-Raf were mutated to Phe constructing a Tyr340Phe/Tyr341Phe mutant as reported in an experimental study, to check the effect of RKIP binding with the N-terminal region of C-Raf (Park et al., 2006). Mutations were also introduced according to the experimental analysis by Wu et al*.* to further confirm the affinity of RKIP ligand-binding pocket residues with the N-terminal region of C-Raf (Wu et al., 2014). Accordingly, three Ala mutations (Asp70Ala, Tyr120Ala, and Tyr181Ala) and two Leu mutations (Pro74Leu and Pro112Leu) were introduced in the WT structural complex and probed for binding affinity of RKIP mutants with C-Raf via molecular dynamics (MD) simulations.

### Analysis of Interaction Dynamics

MD simulations of the near-native docked C-Raf/RKIP structural complex and six mutants computationally constructed were executed to evaluate the stability and dynamic behavior of interacting proteins. The above seven complexes were subsequently prepared for MD simulations with GROMACS v.5.0.6 (GROningen MAChine for Chemical Simulation) software package (Abraham et al., 2015). The AMBER99SB-ILDN forcefield was applied to generate the topology parameters of the structural complexes (Lindorff-Larsen et al., 2010; Venkatesan et al., 2015; Zarei, et al., 2017; Galeazzi et al., 2018). The binary complexes were then surrounded by dodecahedron periodic box of SPCE water molecules (Selent et al., 2013; Venkatesan et al., 2015; Galeazzi et al., 2018; Du et al., 2020). Cl^−^ ions were added to the systems to neutralize it prior to minimization (Supplementary Table S1). Before NVT and NPT equilibration, energy minimization of above systems by steepest descent algorithm (50,000 steps) was performed to remove initial steric clashes. A robust NVT (constant number of particles, volume and temperature) equilibration protocol of 500 ps at 300 K was applied to all systems using a V-rescale thermostat. NVT was followed by achieving the system equilibration under the NPT (constant number of particles, pressure and temperature) ensemble for 500 ps at 1.0 bar. The complex systems were then subjected to 10 ns of production run under constant temperature (300 K) and pressure (1.0 bar). Electrostatics of long range interactions was estimated by PME (Particle Mesh Ewald) algorithm (Darden et al., 1993) and the LINCS algorithm (Hess et al., 1997) was applied to restrain the bond lengths. The MD output was monitored through assessing the stability and behavior of the structural complexes by calculating the Root Mean Square Deviation (RMSD) and Root Mean Square Fluctuation (RMSF) throughout 10 ns of simulation run. Additionally, the dynamics of all systems were scrutinized by visualizing in Visual Molecular Dynamics (VMD) program (Hess et al., 1997) and DS.

### Binding Free Energy Calculations using MM/PBSA and MM/GBSA Method

The free intermolecular binding energy of C-Raf with RKIP and its variants was estimated using Molecular Mechanics/Poisson Boltzmann Surface Area (MM/PBSA) and Molecular Mechanics/Generalized Born Surface Area (MM/GBSA) methodology (Chen et al., 2016; Siebenmorgen and Zacharias, 2020). The binding free energy (BFE) *∆G*_*bind*_ for the complex was calculated according to the below equation for the WT and mutated structural complexes.

$$\Delta G_{bind}=G_{complex}-\left(G_{protein1}+G_{protein2}\right)$$ (1)

The *G*_*complex*_ refers to binding energy of the C-Raf/RKIP structural complex while *G*_*protein1*_ and *G*_*protein2*_ refers to energies of individual proteins within the complex. The free energy of binding with MM/PBSA method was estimated using the *g_mmpbsa* tool of GROMACS (Kumari et al., 2014). In addition, the MM/GBSA approach was applied to identify the essential residues involved in the protein-protein binding interface for providing the per-residue energy decomposition in the WT C-Raf/RKIP complex structure. To evaluate the same, HawkRank scoring function (Feng et al., 2017) incorporated in the HawkDock (http://cadd.zju.edu.cn/hawkdock/) web-server (Weng et al., 2019) was implemented.

### Identification of Druggable Binding Sites

Based on the representative WT structural complex of C-Raf/RKIP, the ANCHOR (http://structure.pitt.edu/anchor/) web-server (Meireles et al., 2010) was employed for identifying the druggable binding sites in the protein-protein interaction complex. Given the structural complex, ANCHOR evaluates the change in solvent accessible surface area (∆SASA) for each side-chain, along with its contribution to the binding energy. It thus facilitates the identification of “hot spots” residues, where small molecule inhibitors have high propensity to bind.

## Results

### Molecular Docking and Molecular Dynamics Simulation Analysis of RKIP With C-Raf

The predicted complex structures from the two web servers were subjected to detailed analysis in DS on the basis of their binding mode and interacting residues.

Knowledge-based protein-protein docking was performed employing HADDOCK web-server to explore the putative binding mode of interaction between C-Raf and RKIP. About 191 structures in four clusters were identified using HADDOCK which represented 95.5% of the water-refined models. HADDOCK score is calculated as a weighted sum of van der Waals intermolecular energy, electrostatic intermolecular energy, desolvation energy, distance restraints energy and buried surface area. Along with HADDOCK score, Z-score is represented as the number of standard deviations from the average a particular cluster is located in terms of score. Negative Z-scores are postulated as being better exemplification of a good HADDOCK cluster. Out of the four clusters, the top two clusters (cluster 3 and cluster 2) were found to have negative Z-scores, while the remaining two clusters (cluster 1 and cluster 4) with positive Z-scores were not considered for further analysis. The cluster three represented the highest negative value in terms of HADDOCK score (−182.3 +/− 8.3) and Z-score (−1.2). The contribution of van der Waals energy and electrostatic energy was observed to be −77.1 +/− 6.0 kcal/mol and −329 +/− 43.8 kcal/mol, respectively. Buried surface area (BSA) criterion was used to evaluate the amount of protein surface not in contact with water. Higher BSA value of 2017.2 +/− 66.3 indicated that the structural complex is compact. Furthermore, RMSD of 0.6 +/− 0.4 was reported to be significantly lower for cluster 3. The four best structural complexes from cluster three were downloaded as PDB files and further examined in terms of their interactions in DS. The third structural complex displayed favorable interactions compared to the remaining three complexes. Residues Tyr340, Tyr341, Trp342, Glu345, Glu348, Arg398, and Lys399 from C-Raf were observed to interact with residues Asp70, Ala73, Lys80, Tyr81, Trp84, His86, Gly110, Tyr120, Tyr181, Glu182 of RKIP in the chosen structural complex (Supplementary Table S2). The interactions were majorly characterized by electrostatic bonds, hydrogen bonds and π-π/π-alkyl bonds.

FFT-based docking program ZDOCK was further used to get a unanimous docking pattern as HADDOCK structural complex. The 10 structural complexes as ZDOCK results were downloaded and analyzed in DS. Two different binding modes were observed between C-Raf and RKIP resulting in two clusters. The complexes from the largest cluster were further investigated on the basis of intermolecular interactions. Accordingly, Complex5 from the largest cluster was observed to make favorable interactions in terms of hydrophobic, electrostatic and hydrogen bonds. Thus, Complex5 was selected as an ideal model from ZDOCK analysis. Residues Tyr340, Tyr341, Trp342, Glu345, Arg398, and Lys414 from C-Raf were observed to interact with RKIP binding pocket residues Ala73, Pro74, Lys80, Tyr81, His86, Gly110, and Tyr181 in Complex5 characterized by hydrogen, hydrophobic and electrostatic interactions (Supplementary Table S2).

**FIGURE 1:**
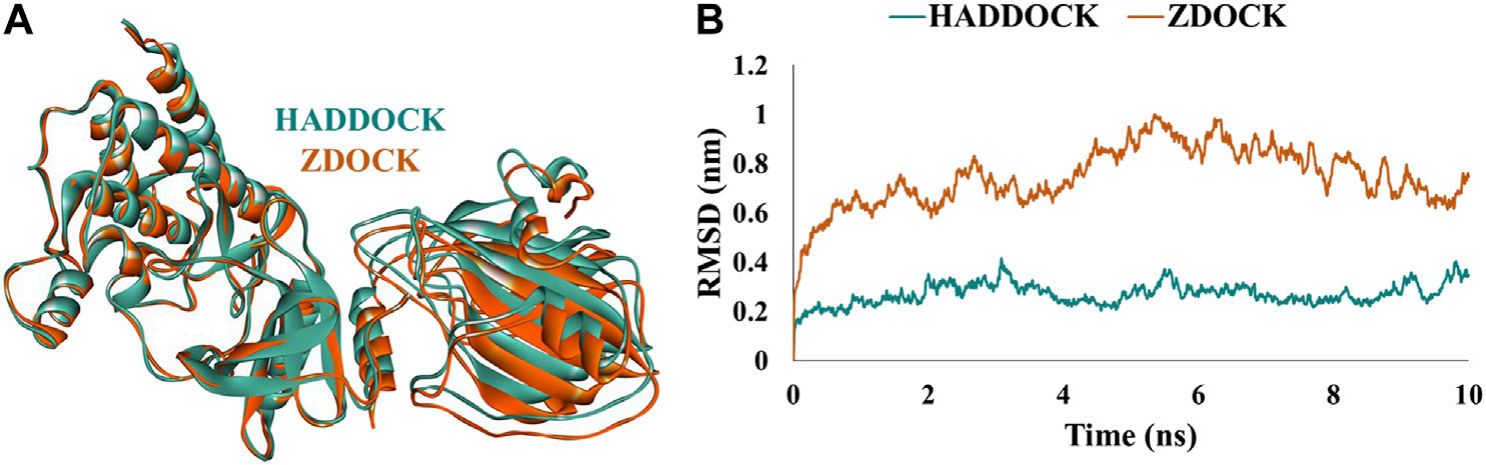
**(A)** Structural alignment and **(B)** comparative backbone root mean square deviation (RMSD) analysis of docked structural complexes generated from HADDOCK (mint green) and ZDOCK (orange) web-servers.

Furthermore, analysis of docked complexes from the above servers showed the occurrence of six C-Raf (Tyr340, Tyr341, Trp342, Glu345, Arg398, and Lys399) and eight RKIP (Asp70, Ala73, Lys80, Tyr81, His86, Gly110, Tyr181, and Glu182) actively interacting amino acid residues observed as common in docked complexes from both servers (Supplementary Table S2). Comparative analysis of the binding modes was performed by superimposing the structural complexes in DS. The generated complexes from the two knowledge-based docking programs superimposed well on each other, thereby displaying a similar binding pattern of interaction between the two proteins (Figure 1A). The complexes were further subjected to MD simulations to assess their stability over a time period of 10 ns by computing the RMSD of backbone atoms. The structural complex generated by HADDOCK program demonstrated an average RMSD of 0.26 nm compared to the average RMSD of 0.74 nm displayed by the complex from ZDOCK docking server (Figure 1B). As a result, the mode of interaction (hereafter referred to as WT complex) displayed by the HADDOCK docking protocol was chosen for subsequent analysis.

### Interaction Dynamics of WT and Mutant C-Raf/RKIP Structural Complexes

**FIGURE 2:**
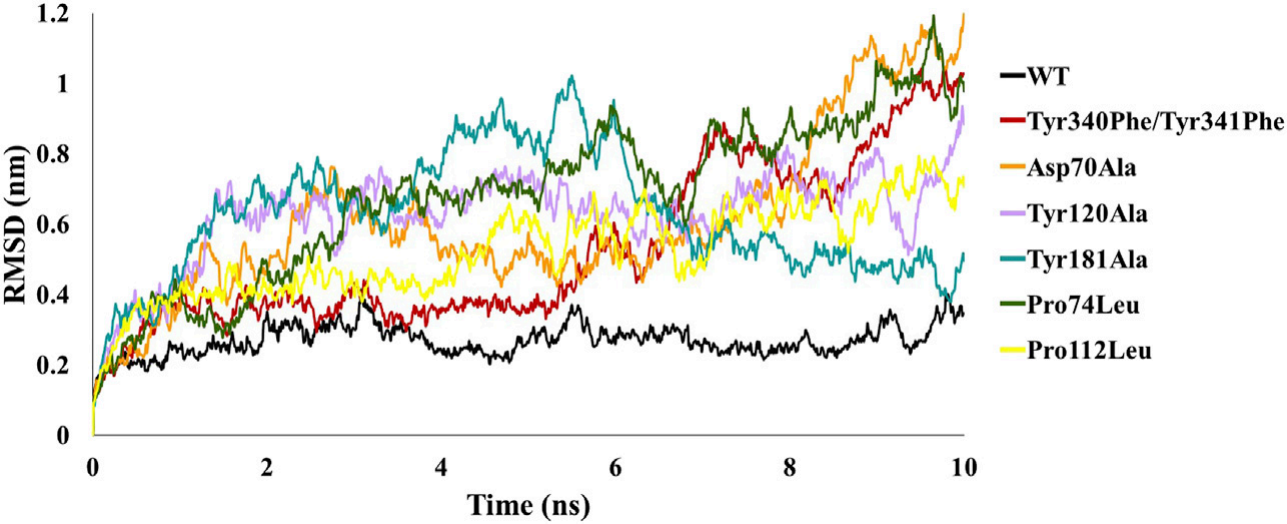
Comparative backbone root mean square deviation (RMSD) analysis of wild-type (WT) structural complex and constructed mutant complexes.

**FIGURE 3:**
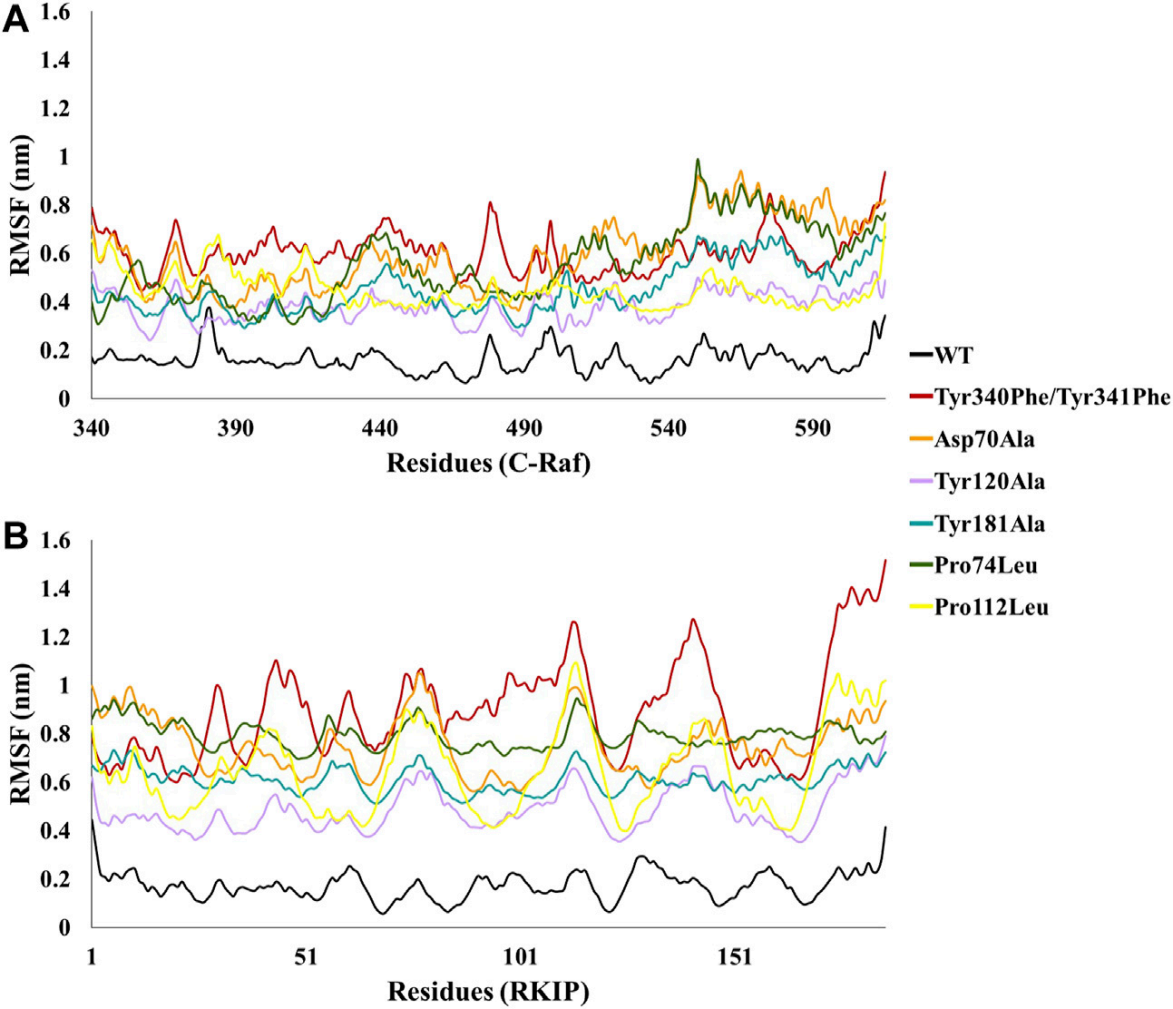
Comparative backbone root mean square fluctuation (RMSF) analysis of wild-type (WT) structural complex and constructed mutant complexes for residues of **(A)** C-Raf and **(B)** RKIP.

In order to analyze the structural consequences upon mutation, the six mutant structures constructed were also subjected to 10 ns of MD simulations. The stability of the structural complexes was assessed by plotting the backbone RMSD values obtained throughout the production run. The inferred RMSD profile for WT complex demonstrated an average RMSD of 0.269 nm as stated above. Similarly, the C-Raf mutant Tyr340Phe/Tyr341Phe residues rendered an average RMSD of 0.527 nm (Figure 2; Table 1). Additionally, the above mutant was observed to abrogate the binding of RKIP with C-Raf. This was consistent with the experimental analysis reported earlier (Park et al., 2006). The three alanine mutations of the RKIP pocket residues Asp70, Tyr120, and Tyr181 were observed to demonstrate an average RMSD of 0.612, 0.628, and 0.622 nm, respectively, (Figure 2; Table 1). The Asp70Ala mutation rendered a substantial increase towards the end of 10 ns, thereby depicting the disruption of regular complex formation. Leucine mutation of RKIP residue Pro74 exhibited an average RMSD of 0.680 nm, while Pro112 mutation displayed an average RMSD of 0.524 nm (Figure 2; Table 1). The Pro74Leu mutant showed a very high fluctuation, while the Pro112Leu mutant displayed a considerable increase as compared to the WT structural complex. The above RMSD analysis suggested that the WT structural complex displayed a stable RMSD throughout 10 ns of production run as compared to the six structural mutants (Figure 2). Subsequent analysis of backbone RMSF by residue indicated an average of 0.165 nm for the WT complex (Figure 3; Table 1). The C-Raf mutant Tyr340Phe/Tyr341Phe demonstrated an average RMSF of 0.721 nm (Figure 3; Table 1). Mutations in RKIP residues rendered an average RMSF in the range of 0.424–0.661 nm (Figure 3; Table 1). From the RMSF graph, it was observed that all the mutants exhibited higher fluctuations across the complex (Figure 3). Additionally, the RMSF of mutated residues in respective mutant complex systems was compared with the RMSF of residues in the WT complex system. Large deviations were observed for all the mutated residues as compared with their RMSF values when in WT complex (Table 2). The overall analysis suggests that mutations on the residues of RKIP could interrupt its complex formation with the N-terminal region of C-Raf.

**TABLE 1:** Molecular dynamics (MD) simulation trajectory analyses for wild-type (WT) and complex systems generated through mutations in conserved interface residues of C-Raf and RKIP.

Sr. No	System	Average backbone RMSD (nm)	Average backbone RMSF (nm)
1	WT C-Raf/RKIP	0.269	0.165
Mutations in C-Raf residues			
2	Tyr340Phe/Tyr341Phe	0.527	0.721
Mutations in RKIP residues			
3	Asp70Ala	0.612	0.661
4	Tyr120Ala	0.628	0.424
5	Tyr181Ala	0.622	0.515
6	Pro74Leu	0.680	0.651
7	Pro112Leu	0.524	0.530

**TABLE 2:** Comparative Root Mean Square Fluctuation (RMSF) analysis of residues in the wild-type (WT) and mutant complex systems.

Sr. No	Residues	RMSF (nm) (WT C-Raf/RKIP complex system)	Mutational change	RMSF (nm) (Mutant complex systems)
1	Tyr340	0.1708	Tyr340Phe	0.7903
2	Tyr341	0.1462	Tyr341Phe	0.7349
3	Asp70	0.0737	Asp70Ala	0.7628
4	Tyr120	0.0875	Tyr120Ala	0.4329
5	Tyr181	0.2244	Tyr181Ala	0.6885
6	Pro74	0.1381	Pro74Leu	0.8456
7	Pro112	0.2069	Pro112Leu	0.9935

**FIGURE 4:**
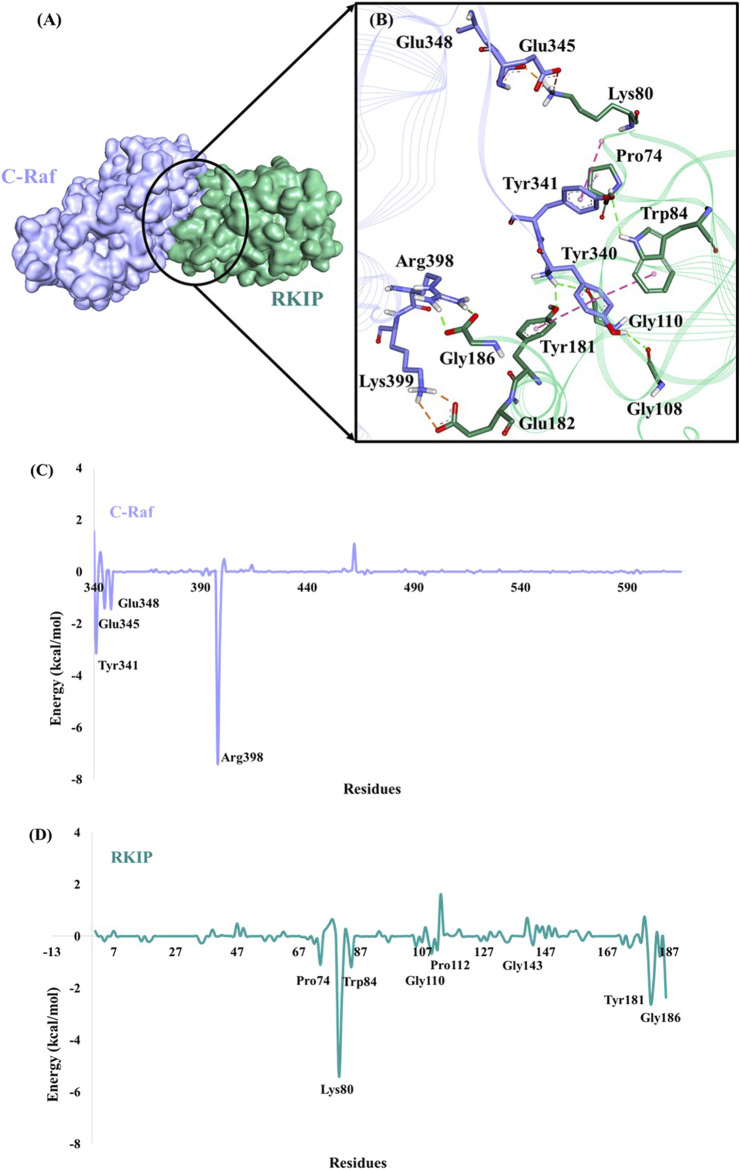
Binding mode of C-Raf/RKIP structural complex and MM/GBSA analysis. **(A)** C-Raf (purple) and RKIP (mint green) shown in surface representation. **(B)**Close-up view of the intermolecular interactions between C-Raf and RKIP. Interacting residues are represented in stick form. Hydrogen, hydrophobic and electrostatic bonds are shown as green, pink and brown dashed lines, respectively. Per-residue energy contribution (kcal/mol) of key residues in **(C)** C-Raf and **(D)** RKIP.

Furthermore, the binding mode for the C-Raf/RKIP interaction (WT) was analyzed by taking a representative pose from the last 5 ns of simulation run. The stable WT complex obtained after MD simulations established strong intermolecular interactions characterized by hydrogen bonds, electrostatic and π-hydrophobic bonds. Residues Tyr340, Tyr341, Glu345, Glu348, Arg398, and Lys399 of C-Raf were observed to be involved in complex formation with residues Pro74, Lys80, Trp84, Gly108, Gly110, Tyr181, Glu182, and Gly186 of RKIP (Figures 4A,B; Table 3). Interactios of C-Raf residues Tyr340, Tyr341, Glu345, Glu348, Arg398, and Lys399 with RKIP residues Lys80, Trp84, Gly110, Tyr181, and Glu182 were found to be consistent both at the beginning (best docked HADDOCK complex) and at the end of the simulation run. The presence of the abovementioned interactions at both times suggest a very stable binding. Moreover, Gly110 and Tyr181 of RKIP were found to interact with C-Raf *via* hydrogen bonds (H-bond) (Table 3). Similar interactions via H-bonds with above residues were reported in a recent study where HIF-1α is shown to interact with RKIP ligand-binding pocket residues (Srivani et al., 2020).

**TABLE 3:** Protein-protein interactions in the proposed C-Raf/RKIP wild-type (WT) structural complex refined through molecular dynamics simulations.

Hydrogen bond interactions					Hydrophobic interactions		Electrostatic interactions	
C-raf		RKIP		Distance	C-raf	RKIP	C-raf	RKIP
Residues	Atoms	Residues	Atoms	(Å)	Residues	Residues	Residues	Residues
Tyr340	H2	Gly110	O	2.76	Tyr340	Trp84	Glu345	Lys80
Tyr340	H2	Tyr181	OH	2.08	Tyr340	Tyr181	Glu348	Lys80
Tyr340	HH	Gly108	O	2.38	Tyr341	Pro74	Lys399	Glu182
Tyr341	OH	Trp84	HE1	2.89	Tyr341	Tyr81		
Arg398	HH12	Gly186	OC1	1.78				
Arg398	HH22	Gly186	OC2	1.79				

The free energy of binding (*∆G*_*bind*_) was calculated for the WT C-Raf/RKIP structural complex as well as for the constructed mutant complexes using MM/PBSA methodology. With the BFE of −174.443 +/− 94.364 kJ/mol obtained for the WT complex, the mutant complexes generated a very low BFE (Table 4). The RKIP binding-pocket mutants Pro74Leu, Pro112Leu, Tyr120Ala, and Tyr181Ala and the C-Raf mutant Tyr340Phe/Tyr341Phe were noticed to have the lowest BFE among the mutant complexes. This could be attributed to the change in conformation thereby impairing the binding. From these values, it can be concluded that the interface residues in the WT structural complex determines a strong stabilization and are essential elements for regular complex formation of RKIP with the N-terminal of C-Raf.

**TABLE 4:** MM/PBSA energy for wild-type (WT) and complexes generated through mutations in conserved interface residues of C-Raf and RKIP.

Sr. No	Protein-protein interaction complex	Binding free energy (∆G_*bind*_) (kJ/mol)
1	WT C-Raf/RKIP	−174.443 ± 94.364
Mutations in C-Raf residues		
2	Tyr340Phe/Tyr341Phe	74.785 ± 101.431
Mutations in RKIP residues		
3	Asp70Ala	−81.406 ± 82.238
4	Tyr120Ala	24.055 ± 74.553
5	Tyr181Ala	36.921 ± 45.577
6	Pro74Leu	170.387 ± 94.431
7	Pro112Leu	118.645 ± 103.452

Moreover, per-residue energy decomposition analysis of the MD simulation-derived equilibrated trajectory of C-Raf/RKIP structural complex was estimated by MM/GBSA implemented in the HawkDock web-server. Our results showed that C-Raf residues Arg398, Tyr341, Lys399, Glu348, and Glu345 along with RKIP residues Lys80, Tyr181, Gly186, Tyr81, Glu182, Trp84, Pro74, and Gly110 are the most critical residues for complex formation (Table 5). From the HawkDock MM/GBSA analysis, it was perceived that Arg398 (C-Raf) and Lys80 (RKIP) contribute majorly to the binding of the complex (Figures 4C,D; Table 5). Additionally, ANCHOR web-server analysis of the MD simulation derived structure of C-Raf/RKIP complex also confirmed Arg398 (C-Raf) and Lys80 (RKIP) as major sites contributing significantly to the binding of the complex (Table 6). Short MD simulations of 10 ns were additionally performed each for Arg398Ala and Lys80Ala mutant complexes in order to check their BFE through MM/PBSA. A significant difference of BFE was noted for Arg398Ala (−57.705 +/− 116.120 kJ/mol) and Lys80Ala (51.747 +/− 101.711 kJ/mol) mutant complexes as compared to the WT C-Raf/RKIP complex (Table 7).

**TABLE 5:** Per-residue energy contribution of five critical residues obtained through HawkDock MM/GBSA analysis for equilibrated simulation trajectory of C-Raf/RKIP structural complex.

C-raf		RKIP	
Residues	Binding free energy (kcal/mol)	Residues	Binding free energy (kcal/mol)
**Arg398**	−7.39	**Lys80**	−5.38
Tyr341	−3.13	Tyr181	−2.59
Lys399	−2.65	Gly186	−2.36
Glu348	−1.45	Tyr81	−2.24
Glu345	−1.42	Glu182	−1.76

*Majorly contributing residues highlighted in bold.

**TABLE 6:** Per-residue energy contribution of five key residues obtained through ANCHOR analysis for equilibrated simulation trajectory of C-Raf/RKIP structural complex.

C-raf		RKIP	
Residues	Binding free energy (kcal/mol)	Residues	Binding free energy (kcal/mol)
**Arg398**	−12.8	**Lys80**	−15.9
Lys399	−9.9	Gly186	−12.1
Glu345	−8.9	Glu182	−10.4
Glu348	−6.4	Lys148	−1.6
Tyr340	−5.8	Tyr181	−1.4

*Majorly contributing residues highlighted in bold.

**TABLE 7:** MM/PBSA energy for wild-type (WT) and complexes generated through mutations of identified druggable “hot spots”.

Protein-protein interaction complex	Binding free energy (∆G_*bind*_) (kJ/mol)
WT C-Raf/RKIP	−174.443 ± 94.364
Arg398Ala	−57.705 ± 116.120
Lys80Ala	51.747 ± 101.711

**FIGURE 5:**
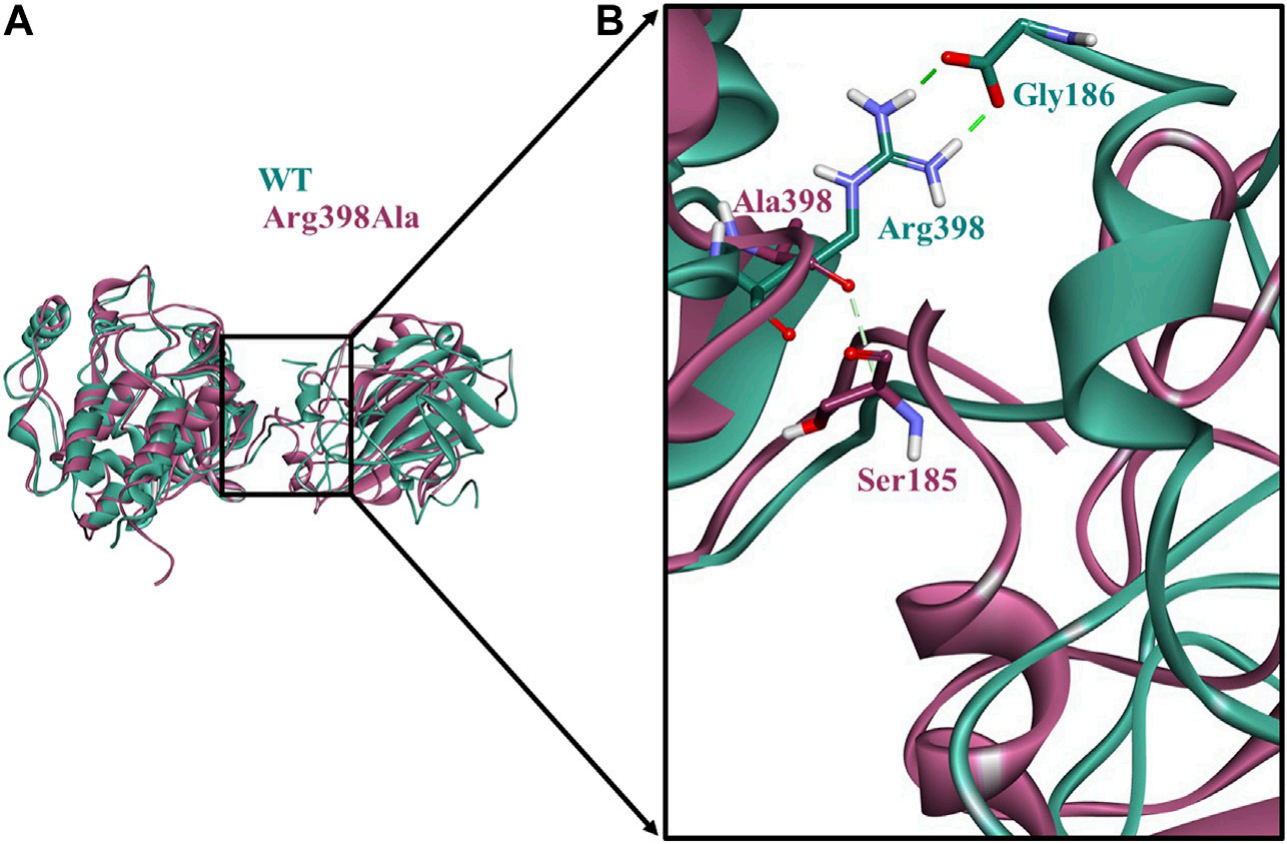
Comparison of the conformational change at the Arg398Ala mutation site as compared with the wild-type (WT) C-Raf/RKIP structural complex obtained through molecular dynamics (MD) simulations.**(A)**The equilibrated native state conformation of WT (mint green) superimposed with the Arg398Ala (mauve) representative snapshot extracted at 180°ns. **(B)** Enlarged view of the conformational change at the Arg398Ala mutation site. Conventional hydrogen bonds are displayed as dark green dashed lines, while carbon-hydrogen bond is shown as a light green dashed line.

**FIGURE 6:**
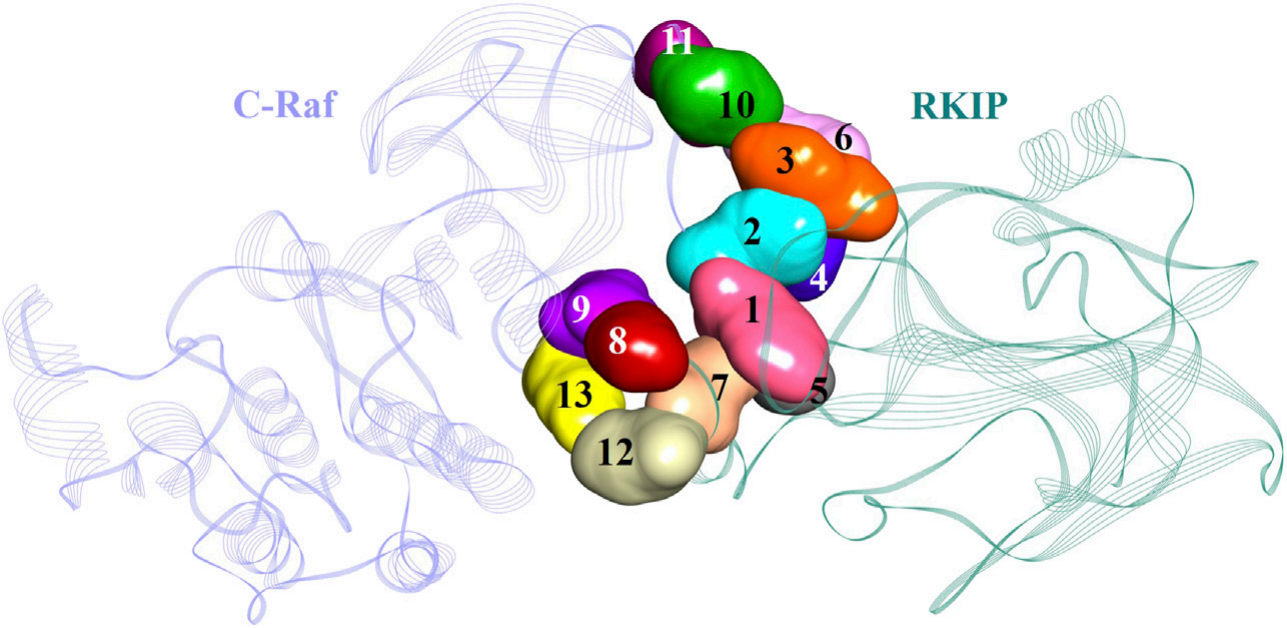
Potential binding sites in the C-Raf/RKIP complex presented as surface with different colors in the structure.

An additional long-time scale MD simulation of 300 ns was performed to investigate the stability of the Arg398Ala mutation starting from the representative structure of WT C-Raf/RKIP structural complex. It was noted that RMSD significantly increased by around 180 ns for the Arg398Ala mutant complex. Moreover, representative snapshot with the largest RMSD value at 180 ns (0.9 nm) for the Arg398Ala complex was extracted and superimposed with the WT representative snapshot (Figure 5A). Significant conformational change occurred in the Arg398Ala mutant complex near the mutation site as compared with the equilibrated native state conformation of the WT C-Raf/RKIP structural complex (Figure 5B). In addition, Ala398 (C-Raf) was observed to form a carbon-hydrogen bond with Ser185 (RKIP) and two conventional hydrogen bonds with Gly186 (RKIP) observed in the WT complex formation were lost. The distance of the carbon-hydrogen bond between Ala398 (C-Raf) and Ser185 (RKIP) of 3.59 Å was greater than the distance of the conventional hydrogen bonds between Arg398 (C-Raf) and Gly186 (RKIP) in WT structural complex (Table 3), thus depicting the importance of Arg398 in regular complex formation with RKIP. Collectively, the C-Raf/RKIP interface binding sites acquired from the WT representative interaction analysis, MM/GBSA analysis and binding sites defined previously by experimental analysis were mapped onto the complex as surface with different colors (Figure 6). Accordingly, the binding sites- Tyr340 (1, pink), Tyr341 (2, light blue), Tyr81 (3, orange), Pro74 (4, dark blue), Gly110 (5, dark grey), Lys80 (6, light pink), and Tyr181 (7, light orange) can be considered as the most essential ones located at the interface of C-Raf/RKIP interaction (Figure 6). Additional literature survey identified anti-leprosy drug, Clofazimine as the C-Raf/RKIP interaction inhibitor in which residues Pro74 and Lys80 were revealed as crucial binding sites (Guo et al., 2018). Moreover, Pranlukast was also identified as a novel ligand, binding to the conserved binding pocket of RKIP and inhibiting its interaction with C-Raf where residues Tyr81 and Tyr181 played a vital role (Sun et al., 2016). Recently, Suramin, initially utilized to treat African sleeping sickness, was identified as C-Raf/RKIP interaction inhibitor binding to residue Tyr181 (Guo et al., 2021). Furthermore, the binding sites- Gly186 (8, maroon), Arg398 (9, violet), Glu345 (10, green), Glu348 (11, dark pink), Glu182 (12, tan), and Lys399 (13, yellow) which are located slightly away from the C-Raf/RKIP interface can be considered as potential allosteric sites (Figure 6). From the above overall analysis, the mapped 13 interface binding sites acquired from the C-Raf/RKIP interaction can be considered as druggable binding sites (“hot spots”) for future designing of small molecule inhibitors that may inhibit the protein-protein interaction between C-Raf and RKIP.

## Discussion

RKIP/PEBP-1 portrays a modulatory role in numerous kinase signaling cascades and was identified as an endogenous regulator of kinases of the RAF/MEK/ERK pathway (Tavel et al., 2012). Besides its role in normal physiological phenomena, dysregulated RKIP expression was observed to contribute significantly to pathophysiological illnesses including Alzheimer’s disease, various cancerous ailments and diabetic nephropathy (Keller et al., 2004; Escara-Wilke et al., 2012; Ling et al., 2014; Farooqi et al., 2015). Interestingly, RKIP was also observed to be a metastasis suppressor (Granovsky and Rosner, 2008; Yesilkanal and Rosner, 2014). Differential regulation of RKIP was also perceived in a variety of human cancers. As a result, RKIP might provide as a valuable indicator for tumor metastases tissues. It is relevant therefore to understand the basis of RKIP inhibition for its application in physiological abnormalities. Developing novel biomarkers will benefit in effective perturbation of RKIP’s involvement in pathological diseases and characterize its ostensibly conflicting roles. The cell sheet migration inhibitor, Locostatin is the only available potent RKIP inhibitor identified till date (Shemon et al., 2009; Rudnitskaya et al., 2012). Locostatin functions as a PPI inhibitor by binding RKIP and abrogates it from interacting with C-Raf (Beshir et al., 2011; Janjusevic et al., 2016). In spite of the accessibility of Locostatin, design of better probes to hinder the association of RKIP with C-Raf for future implications is needed on an urgent basis. However, the 3D structural complex of the PPI between C-Raf and RKIP has not yet been elucidated despite the availability of their individual crystal structures.

Herein, we established the molecular basis of interaction between the two proteins by an *in silico* protocol. A systematic study was designed and a consensus mode of C-Raf/RKIP interaction was obtained by using two knowledge-based docking programs. In particular, the 3D structural model was obtained using a combination of HADDOCK and ZDOCK protein-protein docking web-servers. Similar strategy of integrating multiple docking programs for selection of an ideal binding mode was also implemented in previous studies (Kausar et al., 2013; Venkatesan et al., 2015; Galeazzi et al., 2018; Raghav et al., 2019). The model procured from the HADDOCK knowledge-based docking program was the most reliable as indicated by its stable RMSD obtained throughout the 10 ns of MD production run (Figure 1), negative Z-score, contribution of van der Waals and electrostatic energy. Predominantly, the interactions between the two proteins were characterized by several hydrogen, electrostatic and hydrophobic bonds. Residues Tyr340, Tyr341, and Arg398 of C-Raf were observed to bind Trp84, Gly108, Gly110, Tyr181, and Gly186 of RKIP by six conventional hydrogen bonds (Figure 4, Table 3). Furthermore, the docking/MD protocol was integrated with *in silico* mutagenesis of few conserved interface residues occurring as common amino acids obtained through HADDOCK and ZDOCK docking results (Supplementary Table S2). The impact of mutations on complex formation was verified by additional MD simulations amalgamated with BFE analysis by MM/PBSA methodology. With a BFE of -174.443 kJ/mol for C-Raf/RKIP WT structural complex, the binding energies of constructed mutants were estimated and compared (Table 4).

The two most crucial tyrosines of C-Raf involved in complex formation were mutated to phenylalanine simultaneously. This resulted in repulsion of the two proteins disrupting their bond followed by a substantial upsurge in its stability as observed by its RMSD, RMSF, and BFE of 74.785 kJ/mol (Figures 2, 3; Tables 1, 2, 4). This was in agreement with previously reported experimental study (Park et al., 2006), thus explaining their significant contribution in binding with RKIP via hydrogen bonds. Alanine and leucine mutagenesis of the five conserved residues of RKIP were further analyzed by their diverse RMSD and RMSF plots and differential BFEs (Figures 2, 3; Tables 1, 2, 4). The above mutations were embarked on the basis of the prior RKIP binding study with C-Raf amino acids 1–147 (Wu et al., 2014). However, the influence of the above mutations on the N-terminal region of C-Raf (amino acids 340–615) has not been elucidated yet. The two leucine mutations of Pro74 and Pro112 were observed to abolish the binding between the two proteins and this was also witnessed with their positive binding energies of 170.387 and 118.645 kJ/mol, respectively, (Table 4). Similarly, alanine mutagenesis of Tyr120 and Tyr181 diminished the C-Raf/RKIP interaction resulting in insignificant BFE of 24.055 and 36.921 kJ/mol, respectively (Table 4). From the above analysis, it can be perceived that Pro74, Pro112, Tyr120, and Tyr181 are the most crucial residues for the binding of RKIP with the aforementioned N-terminal region of C-Raf. Alanine mutation of Asp70 also decreased the binding affinity by 46% resulting in BFE of −81.406 kJ/mol (Table 4). The weaker binding affinities of phenylalanine, leucine and alanine mutations in RKIP residues may attribute to the compromised stability and integrity of its ligand-binding pocket with the N-terminal region of C-Raf residues. This explains that the above mutated residues contribute significantly to the regular complex formation of RKIP with C-Raf at the N-terminal.

The snapshot derived from the last 1 ns equilibrated trajectory was subjected to MM/GBSA analysis by HawkDock web-server to characterize the per-residue energy decomposition of important amino acids in complex formation. Moreover, residue contribution in terms of energy and druggable site prediction was also performed by ANCHOR web-server. It was intriguing to note that both servers predicted two residues as indispensable ones for C-Raf/RKIP interaction (Tables 5, 6). Arg398 of C-Raf and Lys80 of RKIP could be regarded as “hot spot” residues along with the above previously identified interface residues and deemed to be druggable sites for future development of novel inhibitors. Using one of the identified “hot spots” as an example, a long-time scale 300 ns MD simulation was performed for the Arg398Ala mutation according to the similar strategy adopted in previous PPI study (Du et al., 2020). A noteworthy difference was noticed near the mutation site when the representative trajectory extracted at 180 ns (RMSD of 0.9 nm) was compared with the representative WT snapshot (Figure 5).

Taken together, comparative protein-protein docking, MD simulations, MM/PBSA and MM/GBSA results revealed vital residues in the interaction of RKIP with C-Raf N-terminal residues Tyr340-Lys615. These 13 residues were mapped as surface with different colors demonstrating their location at the C-Raf/RKIP interface (Figure 6). Accordingly, the binding sites 1–7 located close at the interface can be regarded as active sites while binding sites 8–13 located away from the interface can be considered as allosteric inhibition sites. Identification of RKIP hot spots is imperative in designing anti-RKIP drugs when the aim is to disrupt the C-Raf/RKIP association. Likewise, the acquired information regarding the pivotal amino acids of C-Raf can be utilized in the process of developing C-Raf mimicking peptides for RKIP inhibition as well as for development of novel Raf-1 kinase inhibitors. The present study is the first attempt towards the computational binding analysis of RKIP’s interaction with the N-terminal of C-Raf residues Tyr340-Lys615 employing protein-protein docking approach and MD simulations.

## Conclusion

Targeting the RAF/MEK/ERK pathway represents a potential strategy for the treatment of pathological illnesses including Alzheimer’s disease and cancer. In this work, the interaction of RKIP with the N-terminal of C-Raf (residues Tyr340-Lys615) was investigated by employing two knowledge-based protein-protein docking web-servers which provided a consensus mode of interaction. Docking was followed by refinement of the associated complex by MD simulations. *In silico* mutagenesis of either residues of C-Raf or RKIP that could significantly impact complex formation indicated a lower binding free energy for constructed mutant complexes as compared to the free energy of binding for wild-type C-Raf/RKIP structural complex (−174.443 kJ/mol). A substantial surge in stability was noticed for the mutant complexes as observed from their individual root mean square deviations and fluctuations, thus suggesting that the residues contribute significantly for the regular C-Raf/RKIP interaction. Analysis of equilibrated MD trajectory revealed two residues (Arg398 and Lys80) as quintessential sites contributing to the C-Raf/RKIP interaction. It is intriguing to note that, compared with the equilibrated native conformation, noteworthy conformational and interaction amendment occurred in the Arg398Ala (one of the “druggable hot spots”) near the mutation site. Overall, our model allows for improved understanding of the interactions between the N-terminal region of C-Raf and RKIP. The thirteen binding residues were mapped as surface on the basis of their location onto the C-Raf/RKIP interface, leading to the identification of active (Tyr340, Tyr341, Tyr81, Pro74, Gly110, Lys80, and Tyr181) and allosteric (Gly186, Arg398, Glu345, Glu348, Glu182, and Lys399) protein-protein interaction inhibition sites. This will provide valuable hints to elucidate the structural basis of RKIP binding with C-Raf and help in the effective design of novel inhibitors blocking C-Raf/RKIP interaction.

## Data Availability

The original contributions presented in the study are included in the article/supplementary files, further inquiries can be directed to the corresponding author.
