# Supplementary material for: Exploring the Binding Interaction of Raf Kinase Inhibitory Protein With the N-Terminal of C-Raf Through Molecular Docking and Molecular Dynamics Simulation
Source: Front Mol Biosci. 2021 May 28;8:655035. doi: 10.3389/fmolb.2021.655035 (PMC8194344; doi:10.3389/fmolb.2021.655035)
Supplement: Supplementary file 1 [file DataSheet1.docx]

Supplementary Material

## Supplementary Tables

**Table S1.** Details of the molecular dynamics (MD) simulation systems for wild-type (WT) and constructed mutant complexes.

| **Protein-protein interaction complex system** | **Water**  **box** | **Water**  **model** | **Water**  **molecules** | **Buffer**  **Cl^-^ ions** | **Production**  **time (ns)** |
| --- | --- | --- | --- | --- | --- |
| WT C-Raf/RKIP | Dodecahedron | SPCE | 30926 | 6 | 10 |
| Tyr340Phe/Tyr341Phe | Dodecahedron | SPCE | 30927 | 6 | 10 |
| Asp70Ala | Dodecahedron | SPCE | 30928 | 6 | 10 |
| Tyr120Ala | Dodecahedron | SPCE | 30929 | 6 | 10 |
| Tyr181Ala | Dodecahedron | SPCE | 30929 | 6 | 10 |
| Pro74Leu | Dodecahedron | SPCE | 30925 | 6 | 10 |
| Pro112Leu | Dodecahedron | SPCE | 30925 | 6 | 10 |
| Arg398Ala | Dodecahedron | SPCE | 30933 | 5 | 10 |
| Lys80Ala | Dodecahedron | SPCE | 30934 | 5 | 10 |

**Table S2.** Interacting residues of the C-Raf/RKIP structural complex predicted by HADDOCK and ZDOCK web-servers.

| **C-Raf/RKIP docked**  **structural complex** | **Interacting residues of C-Raf** | **Interacting residues of RKIP** |
| --- | --- | --- |
| **HADDOCK** | **Tyr340**, **Tyr341**, **Trp342**, **Glu345**, Glu348, His369, **Arg398**, **Lys399** | **Asp70**, **Ala73**, **Lys80**, **Tyr81**, Trp84, **His86**, **Gly110**, Tyr120, **Tyr181**, **Glu182** |
| **ZDOCK** | **Tyr340**, **Tyr341**, **Trp342**, **Glu345**, His369, **Arg398**, **Lys399**, Arg401, Lys414 | **Asp70**, **Ala73**, Pro74, **Lys80**, **Tyr81**, **His86**, Gly108, **Gly110**, Pro112, Gly114, Asp144, **Tyr181**, **Glu182**, Leu184, Ser185 |

*Actively interacting residues obtained as common from both servers are highlighted in bold.
